# The complete chloroplast genome of *Camellia grijsii* ‘zhenzhucha’, a variant cultivar with floral aroma

**DOI:** 10.1080/23802359.2021.1959452

**Published:** 2021-08-02

**Authors:** Fengyu Xie, Xinlei Li, Yingkun Sun, Zhaopeng Wen, Hengfu Yin, Jiyuan Li

**Affiliations:** a State Key Laboratory of Tree Genetics and Breeding, Research Institute of Subtropical Forestry, Chinese Academy of Forestry, Hangzhou, China; b College of Landscape and Forestry, Qingdao Agricultural University, Qingdao, China

**Keywords:** *Camelliagrijsii ‘*zhenzhucha’, chloroplastgenome, phylogenetic analysis

## Abstract

*Camellia grijsii* ‘zhenzhucha’ is a variant cultivar from *Camellia grijsii*, which is also called *Camellia grijsii* ‘juhuacha’.*C. grijsii* ‘zhenzhucha’ is an ornamental shrub with a floral aroma, and is oftenused in landscape. To provide genetic information for genetic research, we have sequenced and assembled the complete chloroplast (cp) genome of *C. grijsii* ‘zhenzhucha’ based on the Illumina Hiseq platform. The assembled complete cp genome of *C. grijsii* ‘zhenzhucha’ was 161,478 bp in length with 37.24% GC, including a large single-copy (LSC) region of 59,942 bp, a small single-copy (SSC) region of 17,294 bp, and a pair of inverted repeats (IRs) of 20,293 bp. The cp genome was annotated with 130 functional genes, consisting of 81 protein-coding genes, 45 transporter RNAs, and 4 ribosomal RNAs. To obtain the phylogeny relationship, the cp genome of *C. grijsii* 'zhenzhucha'has been compared with other *Camellia* species, and the results indicate that *C. grijsii* 'zhenzhucha' is closely related to *C. grijsii*. This study provides fundamental information of *C. grijsii* 'zhenzhucha' cp genome, and has an important reference value for the evolutionary analysis.

*Camellia grijsii* ‘zhenzhucha’ also called *C.grijsii* ‘juhuacha’ is older cultivar, and it is widely used in landscaping in southern China and Japan for more than 100 years. It has been reported that *C.grijsii* ‘zhenzhucha’ is a variant cultivar from *C. grijsii*, and they are similar inmorphology, such as white flower with high fragrance and consistent flowering (Guan et al. 2014). But the flower of *C. grijsii* ‘zhenzhucha’ is rose form double, that is very different from the single of *C. grijsii*, so *C. grijsii* ‘zhenzhucha’ has a better ornamental value than *C. grijsii.* As a result of not knowing exact breeding information, we only can deduce that *C. grijsii* ‘zhenzhucha’ is a mutation of *C. grijsii*.


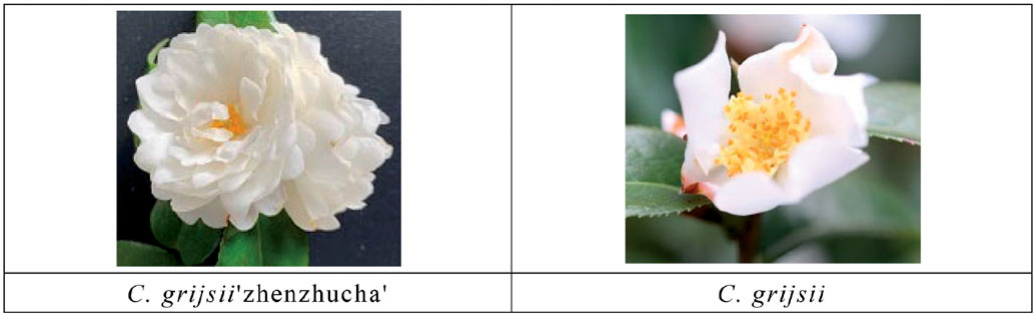


The chloroplast (cp) genome has been extensively used to obtain the knowledge of phylogeny and genetic diversity, especially in species with limited genomic resources, due to the high conservation of sequences, structure and compositions (Wicke et al. 2011). In this paper, we performed the high-throughput sequencing on complete cp genome of *C. grijsii* ‘zhenzhucha’ (NCBI Accession Number: MT916932) and described the assembly and annotation details of the cp genome. Then we inferred the phylogeny of related Camellia species based on the whole cp genomes.

The specimen of *C. grijsii* ‘zhenzhucha’ was deposited at Research Institute of Subtropical Forestry, Chinese Academy of Forestry (http://risfcaf.caf.ac.cn/; Xinlei Li, lixinlei2020@163.com) under the voucher number YL914315. Total DNA was extracted using the MiniBEST plant Genomic DNA Extraction Kit (Takara, Dalian, China). The DNA concentration quality was controlled higher than 20 ng/µL (total mass was higher than 100 mg) by NanoDrop2000 (Thermo Fisher Scientific, USA). To perform sequencing, the Illumina libraries were constructed using TruSeq DNA sample preparation kit (Illumina, San Diego, California, USA).*C. grijsii* ‘zhenzhucha’ was sequenced using Illumina Hiseq 4000 sequencing systems (Illumina, San Diego, California, USA) at Genesky Biotechnologies (Shanghai, China).

During the process of genome assembly, we have carried out strict control and quality evaluation of sequence data.We originally gained 22,992,284 reads and 3,179,400,847 bases. And we retrieved 21,276,562 clean reads and 3,071,352,088 clean bases after quality control using Trimmomatic (Lyu et al. 2019). The clean reads were firstly aligned to the reference genome sequence of *C. japonica* (NCBI Accession Number: NC_036830.1) through Bowtie v2.2.6 (Wang et al. 2018; Cao et al. 2020). The method of assembly and annotation of the cp genome was adapted from Wang et al. (2018). The sequence of cp genome was assembled using Newbler v3.0 (Ye et al. 2017) with the default parameters. Finally, manually confirmed by comparison with the chloroplast genome of *C. japonica* (Xie et al. 2021).

The assembled complete cp genome of *C. grijsii* ‘zhenzhucha’ was 161,478 bp in length with 37.24% GC. It displayed the typical quadripartite structure, including a large single-copy (LSC) region of 59,942 bp, a small single-copy (SSC) region of 17,294 bp, and a pair of inverted repeats (IRs) of 20,293 bp. The cp genome was annotated with 130 functional genes, consisting of 81 protein-coding genes, 45 transporter RNAs, and 4 ribosomal RNAs.

We performed a phylogenetic analysis using fifteen complete cp genomes of *Camellia* species. The conserved protein sequences were extracted for alignment (Wang et al. 2018), MEGA v7.0.14 was used to determine the phylogenetic relationships by the Neighbor-joining method (Kumar et al. 2016). The percentage of phylogenetic trees in which the relevant taxonomic units are clustered in the boot test is shown next to the branch. According to the phylogenetic tree, it was found that *C. grijsii* ‘zhenzhucha’ was closely related to *C. grijsii* with 100% bootstrap support (Figure 1). This indicated that our results supported *C.grijsii* ‘zhenzhucha’ is a cultivar originating from *C. grijsii* (Guan et al. 2014).

**Figure 1.**
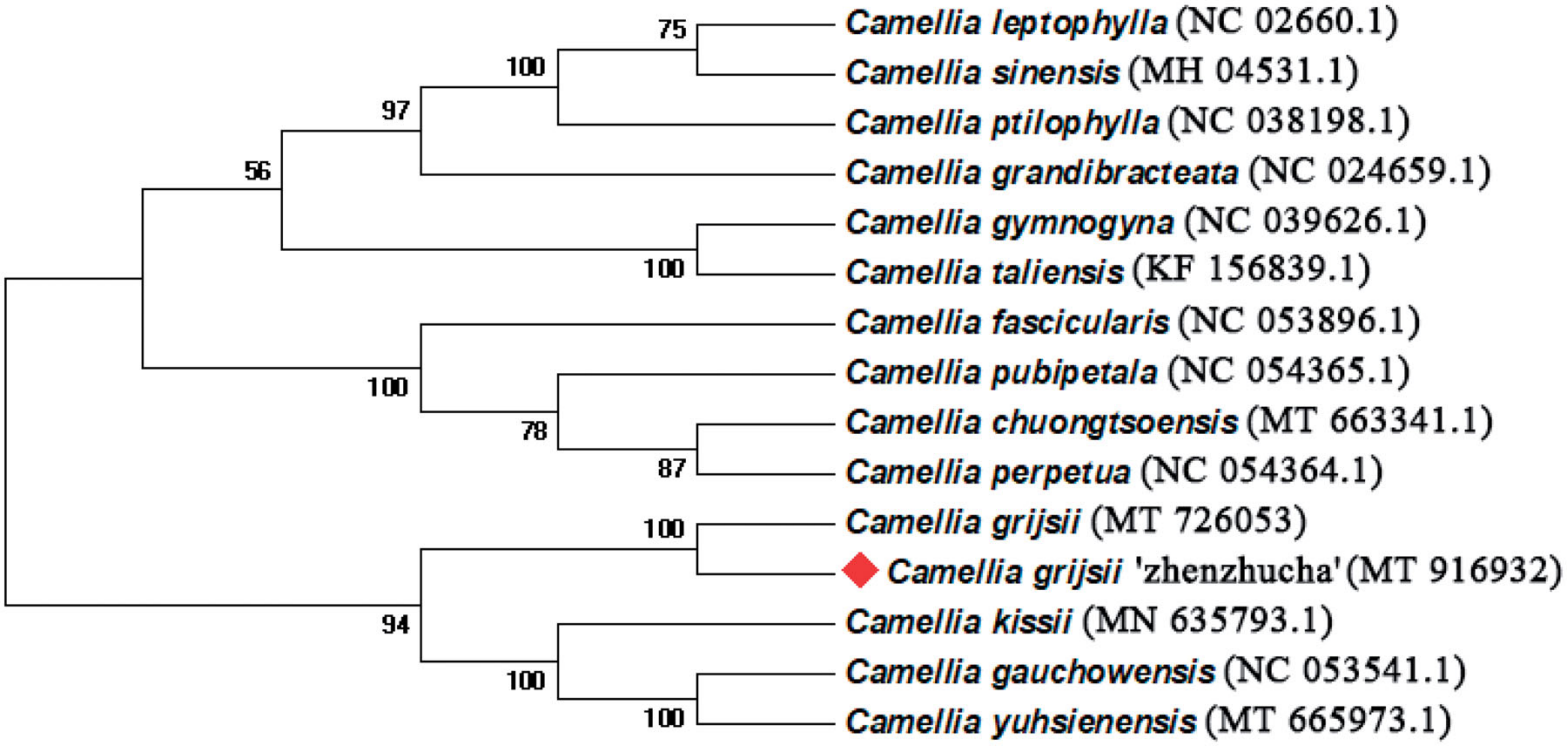
Neighbor-joining phylogenetic tree for *C. grijsii* ‘zhenzhucha’ with other *Camellia* species based on conserved protein sequences of cp genomes.

## Data Availability

The genome sequence data that support the findings of this study are openly available in GenBank of NCBI at [https://www.ncbi.nlm.nih.gov] under the accession No. MT916932. The associated BioProject, SRA, and Bio-Sample numbers are PRJNA743941, SRR15056382, and SAMN20065981 respectively.
